# GABAergic Transmission in the Basolateral Amygdala Differentially Modulates Plasticity in the Dentate Gyrus and the CA1 Areas

**DOI:** 10.3390/ijms21113786

**Published:** 2020-05-27

**Authors:** Rose-Marie Vouimba, Rachel Anunu, Gal Richter-Levin

**Affiliations:** 1 Université de Bordeaux and Institut de Neurosciences Cognitives et Intégratives d’Aquitaine, CNRS, Unité Mixte de Recherche 5287, Allée Geoffroy Saint-Hilaire, CS50023, 33615 PESSAC CEDEX, France; rose-marie.vouimba-siguelnitzky@u-bordeaux.fr; 2 Department of Psychology, University of Haifa, Haifa 3498838, Israel; anunu@univ.haifa.ac.il; 3 Sagol Department of Neurobiology, University of Haifa, Haifa 3498838, Israel; 4 The Integrated Brain and Behavior Research Center (IBBR), University of Haifa, Mount Carmel, Haifa 3498838, Israel

**Keywords:** basolateral amygdala, CA1, dentate gyrus, GABA, metaplasticity, synaptic plasticity

## Abstract

The term “metaplasticity” is used to describe changes in synaptic plasticity sensitivity following an electrical, biochemical, or behavioral priming stimulus. For example, priming the basolateral amygdala (BLA) enhances long-term potentiation (LTP) in the dentate gyrus (DG) but decreases LTP in the CA1. However, the mechanisms underlying these metaplastic effects are only partly understood. Here, we examined whether the mechanism underlying these effects of BLA priming involves intra-BLA GABAergic neurotransmission. Low doses of muscimol, a GABA_A_ receptor (GABA_A_R) agonist, were microinfused into the rat BLA before or after BLA priming. Our findings show that BLA GABA_A_R activation via muscimol mimicked the previously reported effects of electrical BLA priming on LTP in the perforant path and the ventral hippocampal commissure-CA1 pathways, decreasing CA1 LTP and increasing DG LTP. Furthermore, muscimol application before or after tetanic stimulation of the ventral hippocampal commissure-CA1 pathways attenuated the BLA priming-induced decrease in CA1 LTP. In contrast, muscimol application after tetanic stimulation of the perforant path attenuated the BLA priming-induced increase in DG LTP. The data indicate that GABA_A_R activation mediates metaplastic effects of the BLA on plasticity in the CA1 and the DG, but that the same GABA_A_R activation induces an intra-BLA form of metaplasticity, which alters the way BLA priming may modulate plasticity in other brain regions. These results emphasize the need for developing a dynamic model of BLA modulation of plasticity, a model that may better capture processes underlying memory alterations associated with emotional arousing or stressful events.

## 1. Introduction

The term “metaplasticity” [1] is used to describe changes in the ability to induce synaptic plasticity (long-term potentiation (LTP) and long-term depression (LTD) following a priming stimulus activating the synapses. This lasting state change affecting future plasticity is often not apparent in synaptic efficacy following the initial bout of synaptic activity [2]. The priming stimulus may be electrical, biochemical, or behavioral (sensory or emotional experience) [3,4]. For example, studies have shown that both administration of stress hormones (e.g., corticosterone) and behavioral stress result in upregulation or downregulation of LTP, depending on the synapses and the timing of stimulus administration [5,6,7,8,9,10,11,12,13,14]. 

Stress-related alterations of memory are mediated by amygdala–hippocampus interactions, as demonstrated in many human and animal studies [15,16,17,18]. Similar to stress, manipulation of amygdalar activity, particularly the basolateral region of the amygdaloid complex (BLA), can either enhance or impair hippocampal-dependent learning and memory [19,20]. Similarly, we and other groups have reported that BLA activation modulates hippocampal LTP, a synaptic model of memory [21,22,23,24,25]. 

Specifically, in previous studies, we showed that, while activation of the BLA enhances LTP at hippocampal perforant path-dentate gyrus (DG) synapses, it impairs LTP in the ventral hippocampal commissure-CA1 pathway (vHC-CA1) [25,26]. These differential effects of BLA activation on the modulation of hippocampal LTP in the two brain regions have been shown to be mediated by different mechanisms; while activation of glucocorticoid receptors, or β-adrenoceptors, in the BLA modulates synaptic plasticity in the DG, these manipulations do not affect the CA1, implying that other molecular mechanisms are involved in the modulation of synaptic plasticity in this brain region [27]. 

One such alternative mechanism may involve gamma-aminobutyric acid (GABA) neurotransmission, since studies have demonstrated that the BLA contains a powerful inhibitory GABAergic circuit [28,29]. The high concentration of GABA_A_ receptors in the BLA has been suggested to play a critical role in the control of synaptic transmission and plasticity and thereby in stress-induced modulation of memory [15,30,31,32,33,34,35].

Considering the anatomy and the function of the BLA, the distribution of the GABA_A_ receptors in this brain region, as well as the role of the GABA_A_ receptors in memory and anxiety states [36,37], GABAergic modulation of the BLA-hippocampus circuit remains poorly understood. 

The primary purpose of this study was to first examine the involvement of intra-BLA GABAergic neurotransmission in the metaplastic effects of the BLA on hippocampal synaptic plasticity. Secondly, it was to test whether priming the BLA before or after this manipulation leads to intra-BLA metaplastic effects. We demonstrated that, indeed, intra-BLA GABA_A_ receptor activation mediates BLA metaplastic effects on hippocampal plasticity. However, such activation induces intra-BLA form of metaplasticity, which affects the very same BLA-induced metaplasticity in the hippocampus. The results call for the development of a dynamic description of BLA effects on plasticity in other brain areas, a concept that will reflect on developing translational intervention approaches in stress and trauma-related disorders.

## 2. Results

### 2.1. Muscimol Microinfusion into the BLA Increases Excitationin the PP-DG Pathway but Not in the CA1

**Figure 1:**
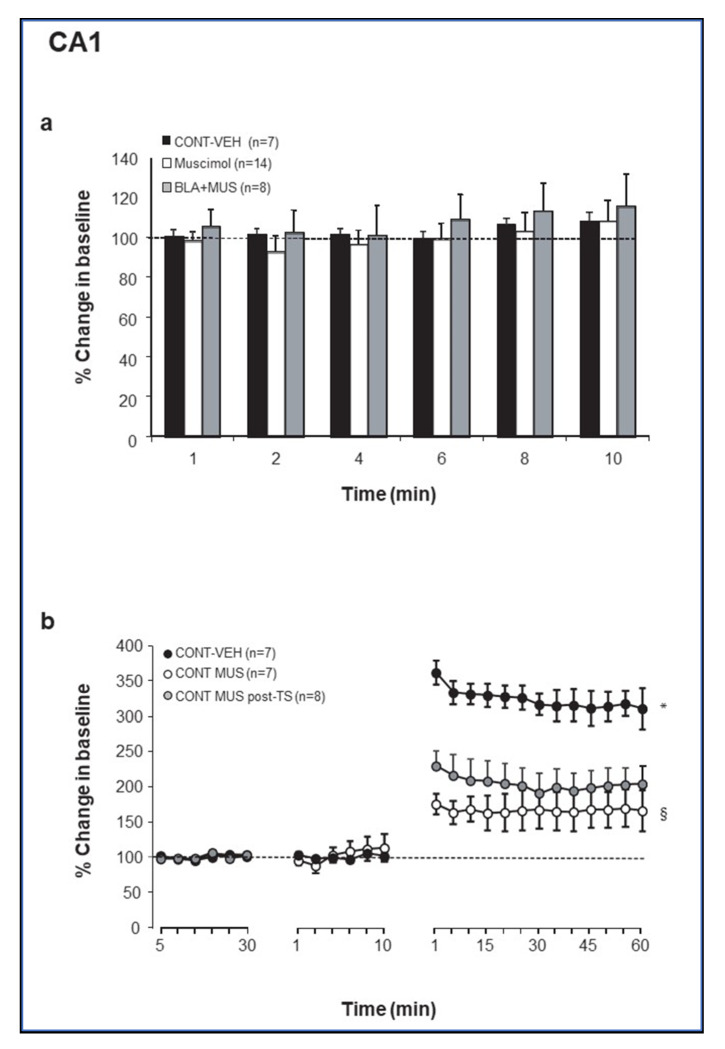
(**a**) Change in baseline excitationin the CA1 subregion following microinfusion of muscimol into the BLA. ns: not significantly different. (Muscimol—*CONT-MUS* and *MUS*-*BLA* (prior to BLA priming) groups). (**b**) Muscimol microinfused either before or after theta-like high-frequency stimulation (TS)-induced LTP suppressed LTP in the CA1. *, Control-VEH is significantly different from both Control-MUS-pre and Control-MUS-post (*p* < 0.05). §, Control-MUS-pre is significantly different from Control-MUS-post (*p* < 0.05).

**Figure 2:**
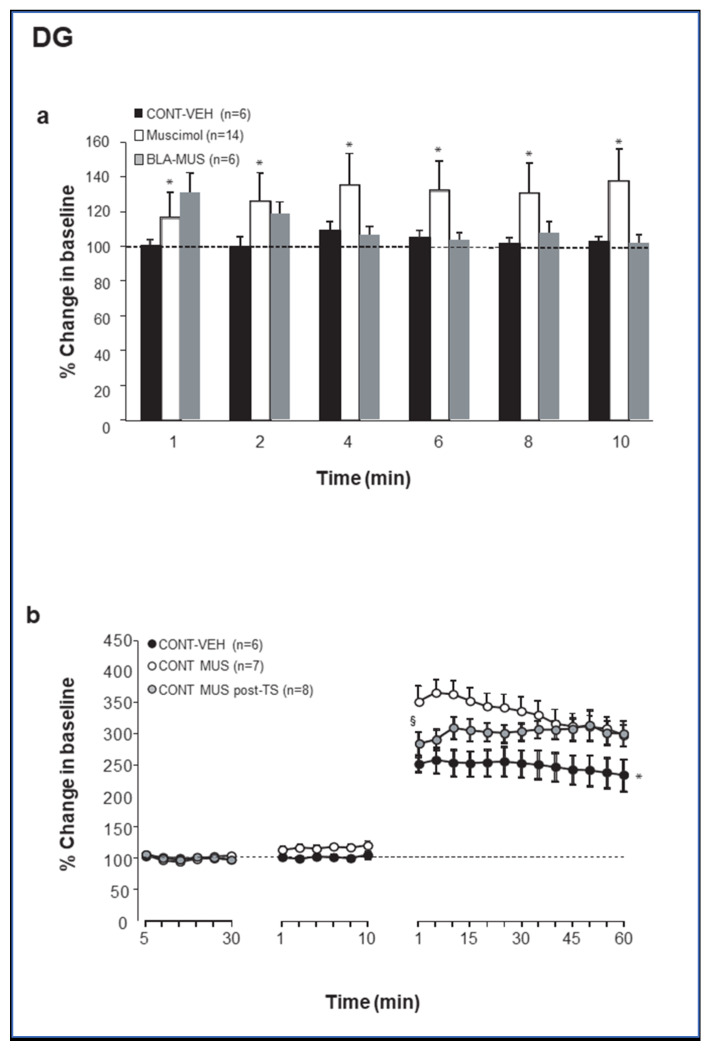
(**a**) Change in baseline excitationin DG following microinfusion of Muscimol in the BLA. ns: not significantly different; * significantly different from both CONT-VEH and CONT-MUS-post (*p* < 0.05). (Muscimol—*CONT-MUS* and *MUS*-*BLA* [prior to BLA priming] groups). (**b**) Muscimol microinfused either before or after TS-induced LTP enhanced LTP in DG but in a slightly different way. Muscimol before enhanced LTP from the start, while muscimol post induced a developing enhancement of the potentiation over time. * Control-MUS is significantly different from both CONT-VEH and CONT-MUS-post (*p* < 0.05); § CONT-VEH is significantly different from both Control-MUS and Control-MUS-post (*p* < 0.05).

To gain insight into GABAergic regulation of BLA modulation of synaptic plasticity in the DG and the CA1, muscimol was microinfused into the BLA, either as a standalone manipulation or 10 min before or after priming of the BLA prior to LTP induction in the hippocampus (see Methods and Figure 5) [38,39]. Muscimol microinfusion into the BLA resulted in no changes in baseline excitation, as measured by the magnitude of CA1 baseline evoked responses (Figure 1a). ANOVA showed that the *MUS* groups (*CONT-MUS* and *MUS-BLA*; see Methods for group designation) were not significantly different from the CONTROL groups (*CONT-VEH* and *BLA-VEH*; *p* = 0.64). In contrast, microinjection of muscimol into the BLA induced a significant increase in baseline excitation in the PP-DG pathway (F (1, 33) = 13.85; *p* < 0.001; Figure 2a). Fisher post hoc tests revealed significant differences between CONTROL (*CONT-VEH* and *BLA-VEH*) and *MUSCIMOL* groups (*CONT-MUS* and *MUS*-*BLA*) one to ten minutes post drug injection (*p* < 0.01 in all cases).

### 2.2. Theta Stimulation Enhances Excitation in the DG but Not CA1 Following Increased GABAergic Transmission in the BLA

Next, we examined the effect of manipulating GABAergic transmission in the BLA on synaptic plasticity in the CA1 and the DG in response to theta stimulation. Muscimol microinfused into the BLA, either 10 min before or immediately after TS, impaired LTP in the vHC-CA1 pathway (percent changes for the last 10 min: *CONT-VEH*: 314.2 ± 22.85%; *CONT-MUS:* 167.25 ± 26.86%; *CONT-MUS-post*: 202.48 ± 24.29%; Figure 1b). Repeated measures ANOVA performed on these data revealed a significant change in the amplitude of vHC-CA1 responses following TS (F (24, 456) = 74.16; *p* < 0.0001), with a significant difference between groups (F (2, 19) = 11.7; *p* < 0.001) and groups–time interaction (F (48, 456) = 18.72, *p* < 0.0001). Fisher post hoc test revealed significant differences between both *CONT-MUS* and *CONT-MUS-post* groups vs. *CONT-VEH* group (*p* < 0.0001 for both) and *CONT-MUS* group vs. *CONT-MUS-post* group (*p* = 0.03).

**Figure 3:**
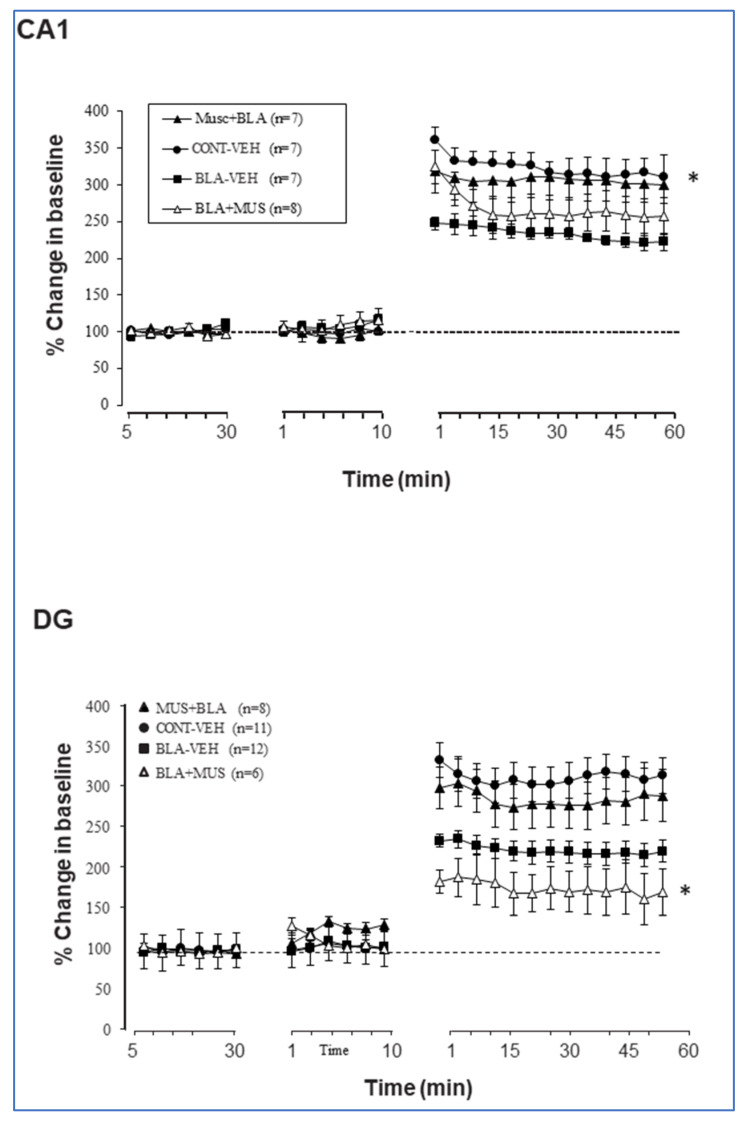
Comparing the effects of repeated (within 10 min) BLA priming to the effects of combined muscimol and BLA priming. In CA1, repeated BLA priming (BLA_BLA) enhanced the LTP reducing effect of a single BLA priming, such that the effect was noticeable already during post-tetanic potentiation * (*p* < 0.05). In the DG, repeated BLA priming (BLA_BLA) reduced the LTP-enhancing effect of BLA priming * (*p* < 0.05), but not to the extent of muscimol post-BLA priming (BLA-MUS). §, BLA_BLA vs. BLA-MUS (*p* < 0.05). It did not reduce the enhancement during the post-tetanic potentiation period except during the LTP phase. For clarity, we did not include the CONT-VEH groups. However, in CA1, the MUS-BLA group was similar to the CONT-VEH group, and in the DG, the level of potentiation towards the end of the BLA-BLA group (at 60 min) was similar to the potentiation level of the CONT-VEH group.

**Figure 4:**
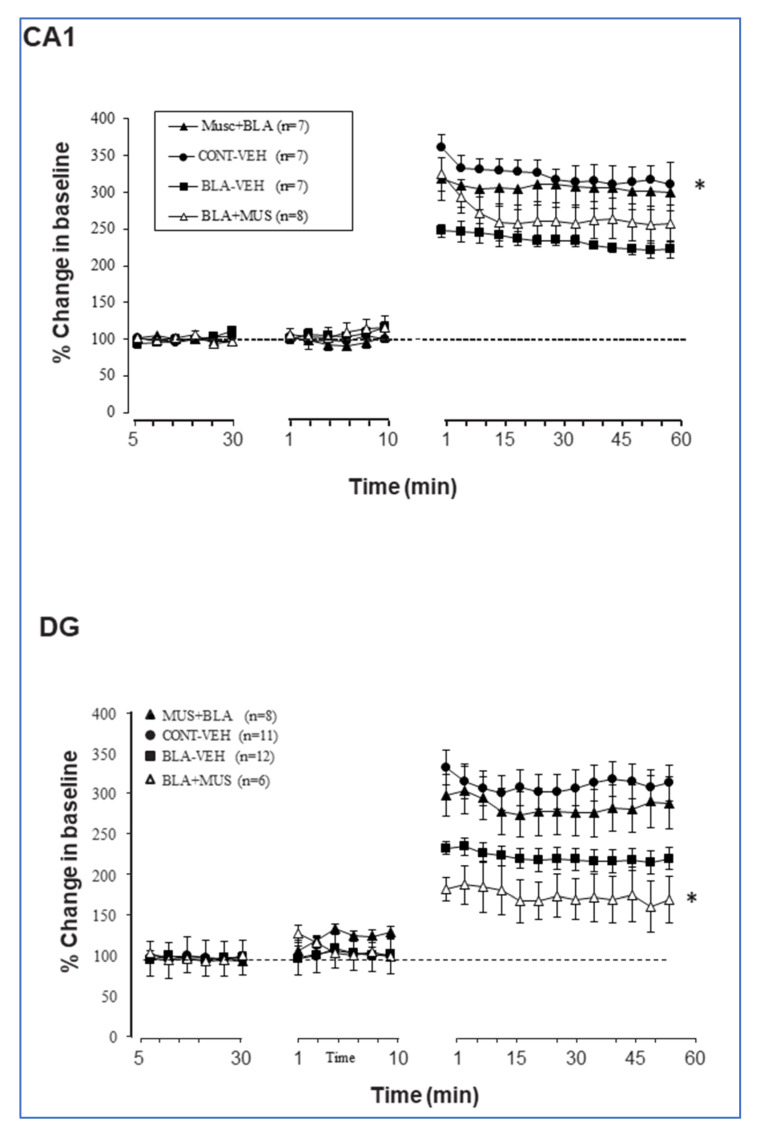
Effects of BLA priming with or without intra-BLA muscimol intervention on LTP induction in the CA1 (*top*) and the DG (*bottom*). In CA1, muscimol prior to BLA priming reduced the inhibitory effect of BLA priming (MUS-BLA significantly different from BLA-VEH, * *p* < 0.05). In the DG, muscimol prior to BLA priming had no effect but muscimol after BLA priming prevented the LTP-enhancing effect of BLA priming. In fact, it reduced LTP even compared to the control (CONT-VEH) group (* *p* < 0.05).

In contrast, muscimol infused into the BLA, either 10 min before or immediately after TS, enhanced LTP in the PP-DG pathway (percent changes for the last 10 min: *CONT-VEH*: 243.8 ± 24.6%; *CONT-MUS:* 305.3 ± 17.39%; *CONT-MUS-post*: 304.9 ± 20.82%; Figure 3). ANOVA revealed a significant difference between groups (F (2, 8) = 6.58; *p* = 0.007; Figure 2b) and groups–time interaction (F (48, 432) = 3.61, *p* < 0.0001). Fisher post hoc test revealed significant differences between both *CONT-MUS* and *CONT-MUS-post* groups vs. *CONT-VEH* group (*p* < 0.01 for both) with no statically significant difference between *CONT-MUS* group vs. *CONT-MUS-post* group (*p* > 0.06), except for post-tetanic responses (1–5 min), which were significantly different (*p* < 0.05). Thus, muscimol microinfusion into the BLA tonically enhanced excitation in DG granular cells but not in CA1 pyramidal cells )see also Figure 3 and Figure 4) and emulated the effects of electrical BLA priming and of stress exposure on both CA1 and DG synaptic plasticity [13,22,26].

### 2.3. Muscimol Application to the Amygdala Increases DG Excitation, Which Is Attenuated if BLA Is Primed

Congruous with previous findings [22,26,27], BLA priming impaired LTP in the vHC-CA1 pathway (percent changes for the last 10 min: *CONT-VEH*: 314.2 ± 22.85%; *BLA-VEH:* 222.07 ± 13.01%), but enhanced LTP in the DG (percent changes for the last 10 min: *CONT-VEH*: 243.8 ± 24.6%; *BLA-VEH*: 322.17 ± 17.62%). ANOVA revealed a significant difference between groups subjected to BLA priming and control groups (*p* < 0.001 in all cases; Figure 4).

When muscimol was microinfused before BLA stimulation, it suppressed the inhibitory effect of BLA activation on CA1 LTP. Thus, the combined effect of muscimol and BLA stimulation suppressed the separate inhibitory effects of either BLA stimulation or muscimol on CA1 LTP (*CONT-VEH*: 314.2 ± 22.85%; *BLA-VEH:* 222.07 ± 13.01%; *BLA-MUS*: 300.88 ± 25.34%). When muscimol was infused after BLA stimulation, LTP was still impaired (last 10 min *BLA-MUS: 254.47* ± *25.1*%). ANOVA performed on these data revealed a significant effect of groups (F (3, 25) = 3.49; *p* = 0.03) and groups–time interaction (F (72, 600) = 2.98; *p* < 0.0001). Post hoc analyses showed a significant difference between *CONT-VEH* group and both *BLA-MUS* and *BLA-VEH* groups (*p* < 0.01) as well as between *BLA-VEH and MUS-BLA* groups (*p* < 0.01). No significant differences were observed between either *CONT-VEH* and *MUS-BLA* or *BLA-VEH BLA-MUS* groups (*p* > 0.05; Figure 4 top). 

In the DG, muscimol microinfused before BLA stimulation had no significant effect on BLA modulation (*BLA-VEH*: 322.17 ± 17.62%; *MUS-BLA*: 296.1 ± 31.9%; *p* = ns). However, when muscimol was microinfused after BLA stimulation, LTP was significantly impaired, even compared to control (percent change from the last 10 min, *BLA-MUS:* 174.04 ± 32.1%). ANOVA performed on these data revealed a significant effect of groups (F (3, 25) = 6.45; *p* = 0.002) and groups–time interaction (F (72, 600) = 5.23; *p* < 0.0001). Post hoc analyses showed significant differences between the *BLA-MUS* group and the three other groups (*p* < 0.01) and no significant difference between *BLA-VEH* and *MUS-BLA* groups (*p* > 0.05; Figure 4 bottom). 

Although BLA and muscimol impacted the CA1 and the DG in a similar way, there was no clear cumulative effect of the joint protocol. In addition, muscimol infused before BLA stimulation suppressed the effect of priming on CA1 LTP but did not significantly reduce the enhancement of DG LTP. When administered after BLA stimulation, muscimol suppressed LTP in both the CA1 and the DG.

We next examined whether priming can be performed more than once to affect metaplasticity (e.g., the modulation of DG or CA1 LTP in response to electrical priming of the BLA) and whether this leads to accumulated priming effect, cancelation of the priming effect, or no change compared to the single priming, as well as how this compares to priming with muscimol. For this purpose, we added a group treated with two repeated BLA priming protocols delivered 10 min apart (*BLA-BLA* group). As seen in Figure 3, the *BLA-BLA* group exhibited stronger impairment in LTP in the CA1 as compared to the *BLA-MUS* group (*p* = 0.015) and the *MUS-BLA* group (*p* < 0.0001). In the DG, the *BLA-BLA* group was significantly different from both the *BLA-MUS* (*p* < 0.0001) and the *MUS-BLA* (*p* = 0.043) groups. However, post-tetanic potentiation (1–15 min post TS) was similar in the *BLA-BLA* group and the *MUS-BLA* groups (*p* > 0.05).

## 3. Materials and Methods 

### 3.1. Subjects 

Experiments were performed on male Sprague-Dawley rats (200–250 g) housed in Plexiglas cages (five rats per cage) and maintained on a free-feeding regimen with a 12:12 h light/dark schedule. All experiments were performed during the light phase of the cycle at least one week after their arrival from the supplier (Envigo, Jerusalem, Israel). All experiments were carried out in accordance with the guidelines of the NIH and approved by the University of Haifa Animal Care and Use Committee. 

### 3.2. Surgery

Rats were anesthetized with urethane (1.2 g/kg, i.p.) in saline (0.9 g/mL) and mounted onto a stereotaxic frame (Stoelting, Wood Dale, IL, USA). The scalp was incised and retracted, and the head position was adjusted to place bregma and lambda in the same horizontal plane. Small burr holes (approximately 1.5 mm diameter) were drilled in the skull for the placement of electrodes and electrode/cannula assembly. During the course of the experiments, body temperature was monitored and maintained at 37 °C ± 0.5 by a regulated heating pad.

### 3.3. Evoked Field Potentials in the CA1 and DG

A recording glass electrode (tip diameter, 2–5 μm; filled with 2 M NaCl) was stereotaxically positioned in the CA1 (4.2 mm posterior to bregma (AP), 2.5 mm from midline (ML), and ~2 mm dorsoventral (DV)) or in the DG (4.2 mm AP, 2.5 mm ML, and ~3.7 mm DV). A bipolar concentric stimulating electrode (125 μm; Kopf, Tujunga, CA, USA) was inserted either in the controlateral ventral hippocampal commissure (vHC: 2 mm AP, 1.5 mm ML, and ~3 mm DV) for activating field potentials in the CA1 or in the ipsilateral perforant pathway (PP: 8 mm AP, 4 mm ML, and ~3 mm DV) for activating field potentials in DG. The dorso-ventral location of the recording and stimulating electrodes was adjusted to maximize the amplitude of the evoked field potentials (EPs). A reference electrode consisting of a 100 μm coated wire was affixed to the skull in the area overlapping the nasal sinus.

### 3.4. Amygdala Activation and Muscimol/Vehicle Injection

An electrode/cannula assembly was inserted in the ipsilateral BLA (3 mm AP, 5.2 mm ML, and 7.6 mm DV) for both drug microinfusion and stimulation. The electrode/cannula assembly was composed of a stimulating electrode made of two 45 µm diameter intertwined platinum-iridium wires (insulated, except at the tip) glued parallel to a 28-gauge stainless steel cannula with a separation of ~300 µm between tips. The assembly was inserted into the brain so that the electrode and the cannula were in a rostro-caudal alignment with the electrode placed rostral to the microinfusion cannula. 

### 3.5. Stimulating and Recording Procedures

CA1 and DG field potentials evoked by single pulses delivered to the vHC or the PP, respectively (0.1 msec rectangular monophasic pulses) were amplified (×1000) by a DAM50 Bio-Amplifier (World Precision Instruments (WPI), Sarasota, FL, US) displayed on an oscilloscope, digitized at 10 kHz (Cambridge Electronic Design Ltd (CED) Cambridge, UK), and saved on disk for off-line analysis (Signal-2 software, Version 1). Baseline responses were established by means of a stimulation intensity (50–200 µA) sufficient to elicit a response representing ~25–30% of the maximal amplitude of the evoked field potentials. 

LTP was assessed by measuring the increase in the population spike amplitude (PS) of the EPs for both DG and CA1. In our previous study [26], we showed that both the PS and the slope of the EPs (for both DG and CA1) follow the same pattern of changes following theta stimulation and amygdala modulation. In this study, because of the early occurrence of the PS in some recordings, the slope was not measurable, and therefore only the PS was analyzed and reported here. 

### 3.6. Drugs and Infusion

To enhance GABA_A_ transmission, the GABA_A_ receptor agonist muscimol (Sigma-Aldrich, St. Louis, MO, USA) was used at a concentration of 0.05 µg/µL. Muscimol was dissolved in physiological saline, which was also used as control. An infusion volume of 0.3 µL (drug or saline) was delivered over a period of 3 min (at a rate of 0.1 µL/min) through a 28-gauge infusion cannula attached to a 10 µL Hamilton micro-syringe (Hamilton Company, Reno, NV, USA) via polyethylene (PE-20) tubing.

### 3.7. Protocols

**Figure 5:**
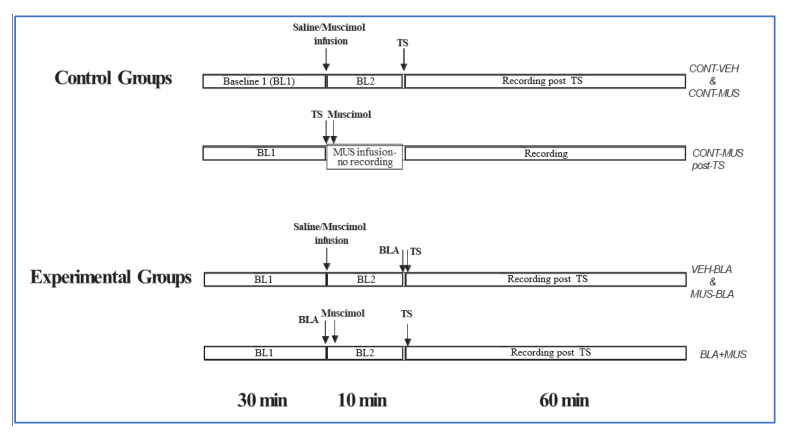
Experimental protocol: The effect of modulating basolateral amygdala (BLA) GABAergic transmission on long-term potentiation (LTP) in ventral hippocampal commissure (vHC)-CA1 and perforant pathway (PP)-dentate gyrus (DG) pathways was examined in two ways: 1. muscimol injection to the BLA without priming the BLA prior to LTP induction in the hippocampus (control groups), and 2. combining muscimol injection and BLA priming (experimental groups) (See Materials and Methods).

To study the effect of manipulating BLA GABA transmission on LTP in the vHC-CA1 and the PP-DG pathways, three baseline and three priming groups of animals were used for each pathway (Figure 5). In the baseline groups, baseline recording of evoked field potential in the CA1/DG was established for 30 min (one pulse every 30 s). At the completion of the baseline, 0.3 μL of saline (*CONT-VEH*) or muscimol *(CONT-MUS)* was infused into the BLA, and a 10 min baseline recording session began after injection of the first 0.1 µL. The 10 min baseline recording was followed by application of theta-like high-frequency stimulation (TS: one set of 5 trains; each train consisted of 5 pulses at 100 Hz; inter-train interval was 200 msec) to the vHC or the PP. After TS, responses to test pulse stimuli were recorded every 30 s for 1 h. In the third group *(CONT-MUS post TS),* muscimol was injected after TS, followed by a 1 h recording.

In the priming groups, the 10 min baseline saline *(BLA-VEH)* or muscimol *(MUS-BLA)* recording was followed by BLA stimulation (1 V, 50 µsec pulse duration, 10 trains of five pulses at 100 Hz; intertrain interval, 200 msec) 30 s before TS to vHC or PP. Responses to low-frequency baseline pulses were then collected every 30 sec for 1h. In the third group *(BLA-MUS)*, the BLA was stimulated prior to the 10 min muscimol infusion, followed by TS to the vHC or the PP and 1 h recording.

**Figure 6:**
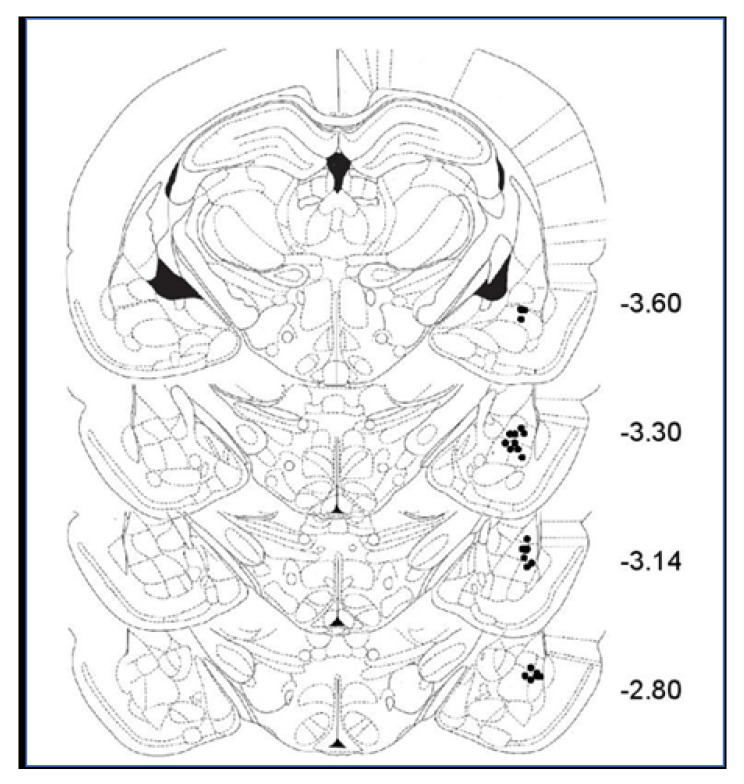
Diagram of coronal sections of the rat brain showing electrode placements in the basolateral amygdala (the figure presents all BLA electrode placements of animals used for Figure 4, but the locations of all electrodes in all animals were verified, and only animals with electrodes in the correct location were included in the analysis).

Figure 6 illustrates the location of stimulation sites in the BLA. The range of the locations of the BLA stimulating electrodes overlapped with those illustrated in our previous studies on BLA [13,26,27,40]. Only animals with a correct positioning of the electrode were included in further analyses.

#### 3.7.1. Muscimol

Muscimol was injected at a volume of 0.3 µL based on preliminary studies and the literature [41,42,43] to ensure its spread was limited to the BLA.

#### 3.7.2. Histology

After completion of the study, animals were transcardially perfused with physiological saline, followed by 10% buffered formalin. Brains were post-fixed in formalin-saccharose 30% solution for at least 3 days, frozen, and cut coronally on a sliding microtome into 50 µm sections. The sections were mounted on a gelatin-coated slide and stained with cresyl violet for microscopic examination of electrode/cannula assembly placement in the BLA.

### 3.8. Data Analysis

The amplitude of the PS of the EPs was expressed as the mean percentage (±SEM) of the individual basal values of animals for each group. Group differences were analyzed by ANOVA and Fisher post hoc tests (Statview).

## 4. Discussion

In the present study, we demonstrated that muscimol, a selective GABAA receptor (GABAAR) agonist, infused into the BLA differentially altered excitation and plasticity in the DG and the CA1. Priming of the BLA with muscimol enhanced excitation and plasticity in the DG but not in the CA1, thereby mimicking the previously reported effect of electrical priming of the BLA [25,26]. However, muscimol also altered the effects of BLA priming on DG and CA1 LTP. These effects were dependent on whether the BLA was stimulated before or after muscimol infusion. The impact of BLA stimulation on CA1 LTP was prevented when muscimol was injected prior to but not after BLA activation, while in the DG, the opposite pattern was observed, i.e., the impact of BLA stimulation on DG LTP was prevented when muscimol was injected after but not prior to BLA activation.

The results support the notion that the BLA modulates hippocampal activity and plasticity in a region-specific manner, with considerably different impact in the DG and the CA1 [25,26]. These findings also support the conception that the way the BLA modulates memory-related processes in other brain areas may be altered by experience and by induced emotional states, a process termed “emotional tagging” [24,25,44]. Thus, behavioral experiences that induce lasting alterations within the BLA can also be expected to affect BLA modulation of memory formation in other brain areas.

We recently demonstrated that inducing a highly selective lasting alteration of BLA activity by way of viral vectors aiming at selectively altering GABAergic synapses at the axon initial segment of principal BLA neurons affected both medial Prefrontal Cortex (mPFC)-dependent behaviors and amenability of the mPFC to induced plasticity [45]. Interestingly, over time, such selective BLA manipulation has led to a form of metaplasticity, which we termed “trans-regional metaplasticity”, i.e., intra-mPFC lasting alterations [45]. These findings indicate that local and selective alteration of activity and plasticity within the BLA may develop into lasting alterations of the emotional circuit.

The present findings further emphasize the potential contribution of activity in GABAergic interneurons to the formation of a shift in the way the BLA modulates memory formation in other brain regions. Sources of GABAergic input to the BLA principal pyramidal-like glutamatergic projection neurons arise from at least two distinct populations of interneurons. The local-circuit neurons, which are scattered throughout the BLA, and the intercalated cell masses (ITCs) surrounding the BLA [46,47,48]. These interneurons can exert tight and powerful control over pyramidal cell activity [49,50]. Specifically, ITCs provide feedforward inhibition in response to excitatory cortical inputs [51], while local circuit interneurons mediate both feedforward and feedback inhibition [52]. Thus, information processing and transmission in the amygdala and particularly within the BLA are determined by both feedback and feedforward GABAergic inhibition [49,53,54]. Furthermore, the amygdala has a high prevalence of GABAA receptors, primarily in the BLA [30], where they are thought to modulate fear, anxiety, learning, and memory by dampening the excitation of the main glutamatergic projection neurons within the BLA [15,55,56]. However, the involvement of BLA GABAergic interneurons may be more complex than simply dampening glutamatergic neurons’ excitation. 

In our previous study, we showed that varying BLA stimulation patterns resulted in differential alterations in serum corticosterone level (CORT), with higher CORT levels being positively correlated with LTP magnitude in DG but not in CA1 [21]. Likewise, muscimol was also reported to increase CORT in a dose dependent manner [57]. In addition, glucocorticoids are known to mediate amygdalar metaplasticity [58] and for being modulators of GABAA receptors in the brain [59]. Karst et al. (2010) [60] reported that corticosterone raises the frequency of mini excitatory postsynaptic currents (mEPSC) of principal cells of the BLA. This increase remained high, even after wash-out of the hormone. Interestingly, previous corticosterone exposure changes the cell’s response to subsequent pulses of corticosterone and leads to a fast and lasting reduction of the mEPSC frequency through a non-genomic GR-dependent pathway. This phenomenon was termed "corticosterone metaplasticity” [60,61].

Inhibition or inactivation of the BLA reduces anxiety and promotes hippocampal-dependent memory and memory processes [62]. In contrast, manipulations that activate the BLA (e.g., electrical or chemical stimulation) enhance the expression of anxiety and decrease hippocampal-dependent memory and memory processes [43,63]. Stressful events have been reported to decrease basal GABAergic activity, leading to an overall increase in BLA neuronal excitation followed by impairment of memory processes [64,65]. However, increased GABAergic transmission may also result in a similar increase in BLA excitation. Studies have reported sustained excitatory effects following prolonged activation of GABAA receptors [66,67]. It has now become increasingly clear that the effects of GABAA receptor activation on the postsynaptic cell can be either inhibitory or excitatory.

Under resting conditions, the amygdala exhibits low neuronal firing due to this particularly high inhibitory tone exerted by the large GABAergic interneuronal network [68]. Either a reduction of GABAergic activity or a direct stimulation of principal pyramidal cells is therefore required to increase excitation of the amygdala. In our experiments, each or a combination of these two different mechanisms can account for the similarity in the impact of muscimol and BLA priming on plasticity in the DG and the CA1. BLA priming directly activates pyramidal neurons but also local interneurons necessary for basic control of excitation. Muscimol-induced excitation may result from muscimol’s direct inhibition of some amygdala interneurons, which partially release the BLA pyramidal cells from the interneurons inhibitory control. This scenario is consistent with the increased expression of immediate-early genes Arc and Homer1a reported following the muscimol injection into the dentate gyrus, indicating increased excitation [69]. However, inhibition of some interneurons may lead to reduced inhibition of other interneurons, thus leading in parallel to some aspects of enhanced inhibition. Muscimol acting through inhibitory GABAARs has been shown to suppress neural activity in the BLA [70,71,72] and consequently to reduce excitatory inputs to inhibitory GABAergic neurons in the central amygdala (CeA). However, studies have shown that muscimol at low concentrations preferentially activates GABACRs, while GABAARs are activated by high muscimol concentrations [73]. The presence of GABACRs was reported in the lateral nucleus (LA) of the amygdala [74]. These receptors were shown to be activated by similar ligands activating GABAARs but with higher binding affinity and slower desensitization rate [73,74].

While GABAARs are localized to the soma of pyramidal cells and interneurons, a study by Cunha and colleagues (2010) [74] suggests that GABACRs are primarily located presynaptically on the axons of interneurons. The location of GABAergic receptors on the synapses is known to affect the functional impact of interneuron activity on the target cells. Thus, GABA receptors in the perisomatic regions provide rhythmic inhibition to pyramidal cells by modulating the generation of somatic action potentials, while those located presynaptically on axons act as autoinhibitors to reduce synaptic GABA release [74,75]. Cunha and colleagues (2010) [74] also showed that activation of GABACRs results in suppression of GABA release and elevation in evoked excitation in the LA, while blocking GABACRs reduces evokes excitation. Moreover, infusion of muscimol into the LA at concentrations that activate GABACRs but not GABAARs enhances fear learning and memory. Therefore, the low dose of muscimol used in the present study, which was previously reported not to induce inactivation [42], may well be expected to result in a relatively selective activation of GABACRs, leading to dishinibition rather than inhibition. Thus, both BLA stimulation and muscimol may have both excitatory and inhibitory impacts on the amygdala [49,53,54,69,73,74]. The end general outcome is probably a result of a balance between these two effects.

It is important to note that a general increased excitation, when combined with more specific and selective increased activity of some interneurons (due to BLA stimulation or to inhibition of inhibition by muscimol), can be expected to lead not to general enhanced effects of the BLA or suppression of its activity but rather to altered processing within the BLA, which modifies the modulation by the BLA of target areas, such as the hippocampus. 

However, BLA and muscimol may also activate different mechanisms, which may lead to similar consequences with respect to the impact on DG and CA1 excitation and LTP. The results obtained with our double priming protocol support such possibility. We showed that two sets of BLA stimulation (double priming) did not yield the same impact on CA1 and DG LTP as the BLA-muscimol protocol (Figure 6). This possibility requires further validation. However, the finding that double manipulation, either by electrical stimulation or by pharmacological manipulation, of the BLA leads to a different outcome compared to a single manipulation indicates that it is of great importance to study the dynamic nature of BLA modulation of plasticity in other brain areas, as it may be critical for understanding emotional memory modulation by the BLA. While most studies have examined the outcome of a single activation of the BLA, accumulating findings indicate that some modes of BLA activation may induce a form of metaplasticity, which would affect the way the BLA responds to a subsequent activating signal. For example, we previously demonstrated that dual BLA priming or repeated exposure to a brief stressful experience results in a form of metaplasticity that prevents the BLA from inhibiting LTP induction in the mPFC [4]. Here, we demonstrate that different modes of BLA activation (e.g., BLA priming or muscimol) may lead to different metaplasticity outcomes. Such differences are likely to contribute, for example, to the large individual variability in response to stress and trauma [2,76]. Future studies will have to develop novel approaches to accommodate the dynamic nature of BLA modulation of memory processes [24,77,78,79].
